# Ga_2_O_3_ Solar-Blind Deep-Ultraviolet Photodetectors with a Suspended Structure for High Responsivity and High-Speed Applications

**DOI:** 10.34133/research.0546

**Published:** 2024-12-11

**Authors:** Xiaoxi Li, Zhifan Wu, Yuan Fang, Shuqi Huang, Cizhe Fang, Yibo Wang, Xiangyu Zeng, Yingguo Yang, Yue Hao, Yan Liu, Genquan Han

**Affiliations:** ^1^ Hangzhou Institute of Technology, Xidian University, Hangzhou 311200, China.; ^2^ School of Microelectronics, Xidian University, Xi’an 710071, China.; ^3^ State Key Laboratory of ASIC and System, Shanghai Institute of Intelligent Electronics & Systems, School of Microelectronics, Fudan University, Shanghai 200433, China.; ^4^ Shanghai Synchrotron Radiation Facility (SSRF), Zhangjiang Lab, Shanghai Advanced Research Institute, Chinese Academy of Sciences, Shanghai 201204, China.

## Abstract

The wide-bandgap semiconductor material Ga_2_O_3_ exhibits great potential in solar-blind deep-ultraviolet (DUV) photodetection applications, including none-line-of-sight secure optical communication, fire warning, high-voltage electricity monitoring, and maritime fog dispersion navigation. However, Ga_2_O_3_ photodetectors have traditionally faced challenges in achieving both high responsivity and fast response time, limiting their practical application. Herein, the Ga_2_O_3_ solar-blind DUV photodetectors with a suspended structure have been constructed for the first time. The photodetector exhibits a high responsivity of 1.51 × 10^10^ A/W, a sensitive detectivity of 6.01 × 10^17^ Jones, a large external quantum efficiency of 7.53 × 10^12^ %, and a fast rise time of 180 ms under 250-nm illumination. Notably, the photodetector achieves both high responsivity and fast response time simultaneously under ultra-weak power intensity excitation of 0.01 μW/cm^2^. This important improvement is attributed to the reduction of interface defects, improved carrier transport, efficient carrier separation, and enhanced light absorption enabled by the suspended structure. This work provides valuable insights for designing and optimizing high-performance Ga_2_O_3_ solar-blind photodetectors.

## Introduction

Solar-blind (200 to 280 nm) deep-ultraviolet (DUV) photodetectors offer marked advantages such as low background noise, all-weather operation, and a high signal-to-noise ratio [1,2]. In recent years, DUV photodetectors have attracted increasing attention due to their vast application potential in areas such as missile tracking, flame detection, and ozone-hole monitoring [3]. Compared to traditional Si-based solar-blind DUV photodetectors with vacuum multiplier tube structure, those based on wide-bandgap (*E*_g_) semiconductors offer notable advantages, including simpler structures, smaller sizes, higher detection sensitivity, low operation voltages, and ease of integration [4]. Extensive research has been conducted on wide-bandgap semiconductors, including GaN (*E*_g_ = 3.4 eV), 4H-SiC (*E*_g_ = 3.2 eV), AlGaN (*E*_g_ = 3.4 to 6.2 eV), ZnMgO (*E*_g_ = 3.4 to 7.8 eV), diamond (*E*_g_ = 5.5 eV), boron nitride (BN) (*E*_g_ = 6.4 eV), and Ga_2_O_3_ (*E*_g_ = 4.6 to 4.9 eV) [5,6]. However, the bandgaps of GaN and 4H-SiC are too narrow to effectively cover the solar-blind spectral range, and the complex growth techniques required for AlGaN and ZnMgO limit their use for DUV detection. Furthermore, the maturity of BN and diamond materials is relatively low, and the high cost of diamond poses challenges for its widespread application in DUV photodetectors. Among these wide-bandgap semiconductors, Ga_2_O_3_ stands out for solar-blind DUV photodetectors due to its intrinsic solar blindness, high thermal stability, high absorption coefficient, excellent radiation hardness, large breakdown field, and availability of high-quality crystal [7,8]. Moreover, the high-quality Ga_2_O_3_ material has been utilized to fabricate various device structures for solar-blind DUV photodetectors, including planar metal–semiconductor–metal (MSM) types, Schottky diodes, p–n diodes, and van der Waals heterostructure devices [9].

Recent efforts have focused on enhancing the photoelectric performance of Ga_2_O_3_ photodetectors, particularly targeting key parameters such as responsivity (*R*) and response time [10]. For instance, Shen et al. developed an MSM-structured Ga_2_O_3_ solar-blind DUV photodetector by optimizing the annealing process. By thermally annealing the material to repair surface defects and suppress carrier trapping, the device achieved a fast response time of 41 ms but a low photoresponsivity of 0.01 A/W [11]. Additionally, Tang and colleagues [12] fabricated a Ga_2_O_3_ solar-blind DUV heterostructure photodetector with zero-dimensional (0D) platinum quantum dots deposited on the Ga_2_O_3_ channel. This structure enhanced DUV light absorption and the injection of photogenerated carriers, resulting in a responsivity of 4.49 A/W and a response time of 0.35 s. Oh et al. [13] further reported a Ga_2_O_3_ solar-blind DUV photodetector with transparent and conductive graphene electrodes, where the modulation of the contact barriers at the graphene/Ga_2_O_3_ interface allowed the device to reach a high responsivity of 29.8 A/W. However, studies indicate that while high responsivity in these devices is often attributed to surface states and defects, these same characteristics can increase recombination rates of photogenerated carriers, ultimately leading to longer response times [14]. Despite progress in this area, optimizing both responsivity and response time simultaneously remains a major challenge for current Ga_2_O_3_ photodetectors. Therefore, the development of innovative Ga_2_O_3_ photodetector structures is urgently needed.

In this work, Ga_2_O_3_ solar-blind DUV photodetectors with a suspended structure were proposed and constructed. The photodetector exhibits a high *R* of 1.51 × 10^10^ A/W, a sensitive detectivity ($D^{*}$) of 6.01 × 10^17^ Jones, a large external quantum efficiency (*EQE*) of 7.53 × 10^12^ %, and a fast rise time (*τ*_r_) of 180 ms under 250-nm illumination. Notably, the Ga_2_O_3_ photodetector demonstrates both a high *R* of 1.51 × 10^10^ A/W and a rapid response time of 180 ms under ultra-weak power intensity (*P*_in_) excitation of 0.01 μW/cm^2^. This improvement is attributed to the combined effects of reduced interface defects, enhanced carrier transport, efficient carrier separation, and increased light absorption enabled by the suspended structure. These findings offer a promising approach for achieving high responsivity and high-speed performance in Ga_2_O_3_-based photodetectors, advancing their potential for practical applications.

## Results and Discussion

**Fig. 1.**
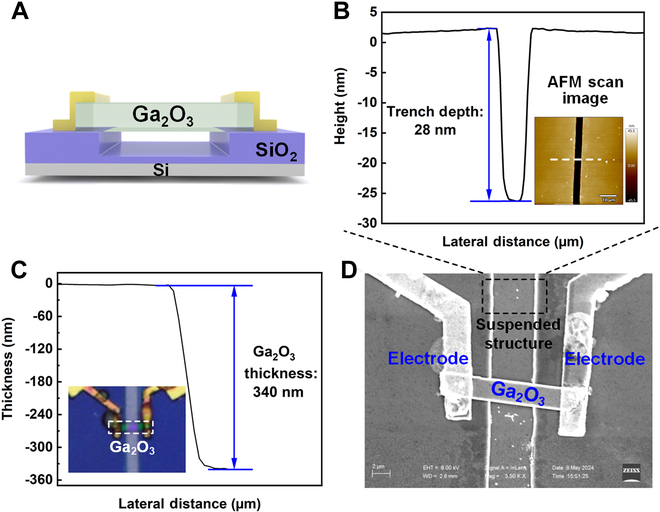
Characterization of the morphology and height of Ga_2_O_3_ devices. (A) Schematic illustration of the Ga_2_O_3_ photodetectors with a suspended structure. (B) Depth of the etched trench. Inset: Surface morphology image of the trench. (C) Thickness of the Ga_2_O_3_ channel. Inset: Optical microscopic image of the fabricated Ga_2_O_3_ photodetector. (D) SEM image of the Ga_2_O_3_ device with a suspended structure.

Figure 1A shows a schematic diagram of the Ga_2_O_3_ photodetector with a suspended structure. The corresponding scanning electron microscope (SEM) image of the fabricated Ga_2_O_3_ photodetector was shown in Fig. 1D, clearly showing the suspended structure and the overall morphology of the device. The Ga_2_O_3_ channel dimensions were precisely measured, with a length of 10.2 μm and a width of 2.6 μm, ensuring the appropriate aspect ratio for optimal device performance. To create the suspended structure, reactive ion etching (RIE) equipment was employed, utilizing CF_4_ and Ar gases as etching agents. This process enabled the precise definition of the trench beneath the Ga_2_O_3_ channel. Following the etching process, the trench depth was measured using an atomic force microscope (AFM), as shown in Fig. 1B. The AFM results revealed a well-defined trench with a depth of 28 nm and smooth, straight sidewalls, which are crucial for maintaining mechanical stability and enhancing the optoelectrical properties of the suspended structure. In addition to trench characterization, the thickness of the Ga_2_O_3_ channel was measured using AFM, as depicted in Fig. 1C, where the thickness of Ga_2_O_3_ film was found to be 340 nm. Besides, the optical microscopy image of the fabricated Ga_2_O_3_ photodetector with a suspended structure was displayed in the inset of Fig. 1C. It can be found that the Ga_2_O_3_ channel was perfectly transferred to the top of the suspended structure without introducing notable mechanical deformation or damage. Furthermore, the surface morphology of the Ga_2_O_3_ channel was characterized using AFM, with the results shown in Fig. S1. The surface was found to be highly uniform, with a root mean square roughness of 2.04 nm. Such a low roughness indicates minimal surface defects, which is essential for reducing carrier recombination and improving the overall responsivity and speed of the photodetector.

**Fig. 2.**
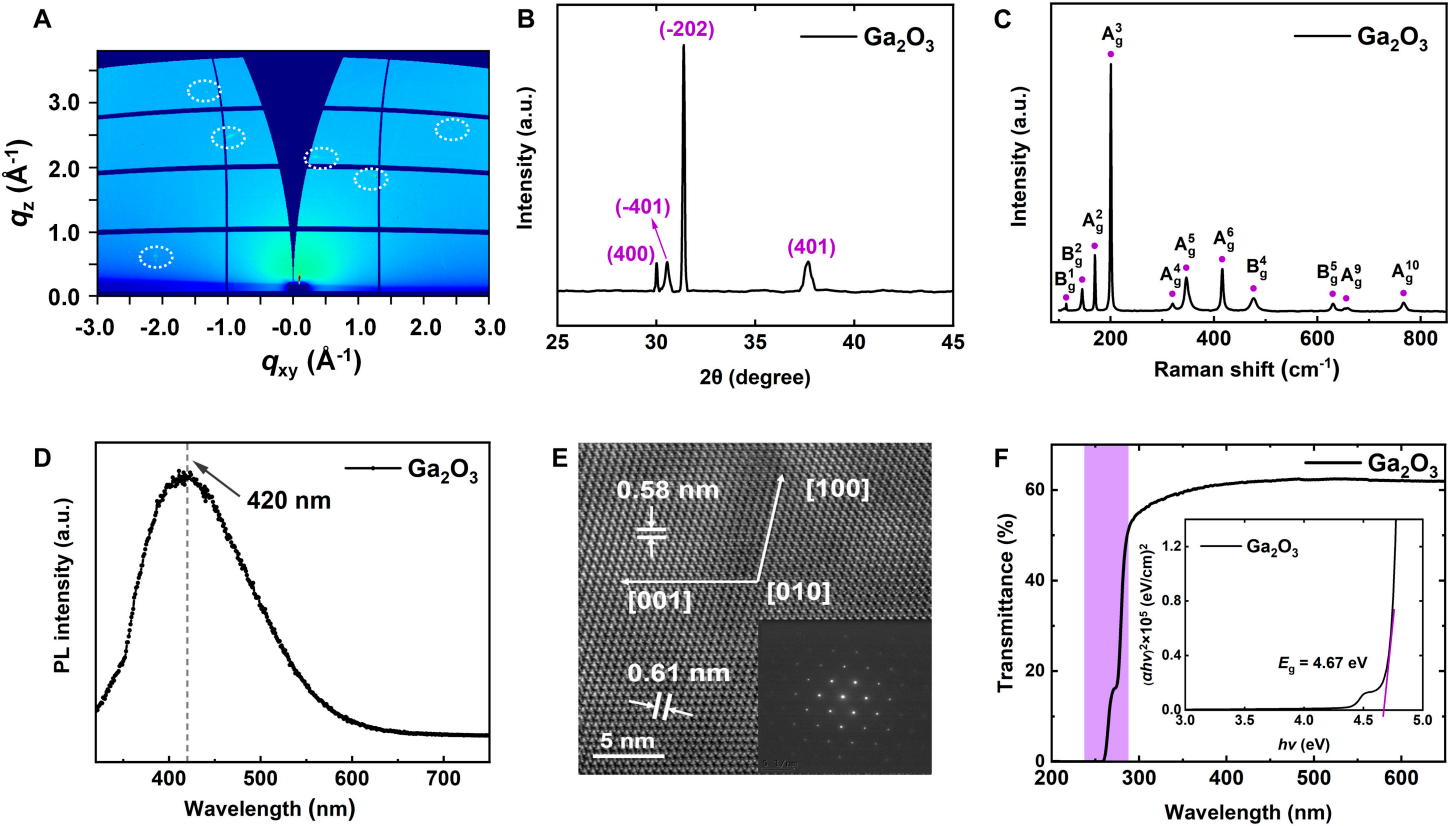
Material characterizations of Ga_2_O_3_ material. (A) 2D-GIXRD pattern of Ga_2_O_3_ material. (B) Integrated 1D-GIXRD spectrum of Ga_2_O_3_ material. (C) Raman spectrum of Ga_2_O_3_ material. (D) PL spectrum of Ga_2_O_3_ material. (E) HR-TEM image of Ga_2_O_3_ material. Inset: SAED pattern of Ga_2_O_3_ material. (F) Transmittance spectrum of Ga_2_O_3_ material. The inset displays the curve of (*αhν*)^2^ versus *hν* used to extract the bandgap of Ga_2_O_3_.

Next, comprehensive physical characterizations of the Ga_2_O_3_ channel were performed to assess its structural and optical properties. Synchrotron-based grazing incidence x-ray diffraction (GIXRD) measurements were initially conducted to evaluate the crystalline structure of the Ga_2_O_3_ channel. Figure 2A shows the 2D-GIXRD pattern, where the presence of sharp and well-defined diffraction spots indicates the high crystalline quality of the Ga_2_O_3_ channel, signifying its superior structural quality [15]. Following this, the azimuthally integrated 1D-GIXRD spectrum was extracted from Fig. 2A, as shown in Fig. 2B. This spectrum reveals diffraction peaks corresponding to the monoclinic *β*-phase of Ga_2_O_3_, which are in agreement with the standard PDF cards (No. 41-1103) [16]. The peaks observed at 30.1°, 30.5°, 31.5°, and 37.4° were indexed to the (400), (−401), (−202), and (401) crystal planes, respectively, confirming the material’s monoclinic structure. Notably, the absence of any impurity-related peaks further demonstrates the high phase purity and crystalline quality of the Ga_2_O_3_ channel. To further explore the optical properties, Raman spectroscopy was performed with a 532-nm laser excitation. The resulting Raman spectrum, shown in Fig. 2C, exhibits distinct peaks in the range of 100 to 850 cm^−1^. Eleven characteristic Raman modes were identified at 114, 145, 169, 200, 320, 346, 416, 477, 631, 658, and 767 cm^−1^, corresponding to $B_{g}^{1}$, $B_{g}^{2}$, $A_{g}^{2}$, $A_{g}^{3}$, $A_{g}^{4}$, $A_{g}^{5}$, $A_{g}^{6}$, $B_{g}^{4}$, $B_{g}^{5}$, $A_{g}^{9}$, and $A_{g}^{10}$ vibration modes, respectively [17]. These results are consistent with previously reported data for Ga_2_O_3_, reinforcing the excellent single-crystalline quality of the channel. In addition, photoluminescence (PL) spectroscopy with a 325-nm laser excitation was performed, as shown in Fig. 2D. A prominent PL emission peak was observed at around 420 nm, corresponding to UV-blue emission. The strong intensity of this peak is indicative of high crystallinity and a low concentration of oxygen vacancies and defects within the Ga_2_O_3_ channel [18], further supporting the high-quality fabrication of the material. High-resolution transmission electron microscopy (HR-TEM) was also employed to explore the morphology and crystal structure in more detail. Figure 2E shows a cross-sectional HR-TEM image of the Ga_2_O_3_ channel, where the observed lattice fringe spacings of ~0.58 nm and ~0.61 nm correspond to the (001) and (200) atomic planes, respectively [19]. This confirms that the Ga_2_O_3_ channel adopts a monoclinic crystal structure, imaged along the [010] zone axis. Moreover, the selected-area electron diffraction (SAED) pattern, inset in Fig. 2E, further corroborates the high symmetry and excellent single-crystal quality of the Ga_2_O_3_ channel [20]. Finally, the optical transmission properties of the Ga_2_O_3_ channel were examined. Figure 2F presents optical transmission spectrum measured over the range of 200 to 650 nm. The Ga_2_O_3_ channel demonstrates high transmittance in the visible light range and a sharp absorption edges in the DUV region, indicating good optical quality and a low density of deep-level defects. Additionally, *E*_g_ can be obtained by Tauc plot formula: (*αhv*)^2^ = C(*hv* − *E*_g_), where *α* is the absorbance coefficient, *h* is the Planck’s constant, and *v* is the frequency of the excitation light. The calculated bandgap was 4.67 eV, which aligns closely with previously reported values for Ga_2_O_3_ [21]. These findings collectively highlight the excellent structural and physical properties of the Ga_2_O_3_ channel, underscoring its potential for high-performance applications in DUV photodetectors and other optoelectronic devices.

**Fig. 3.**
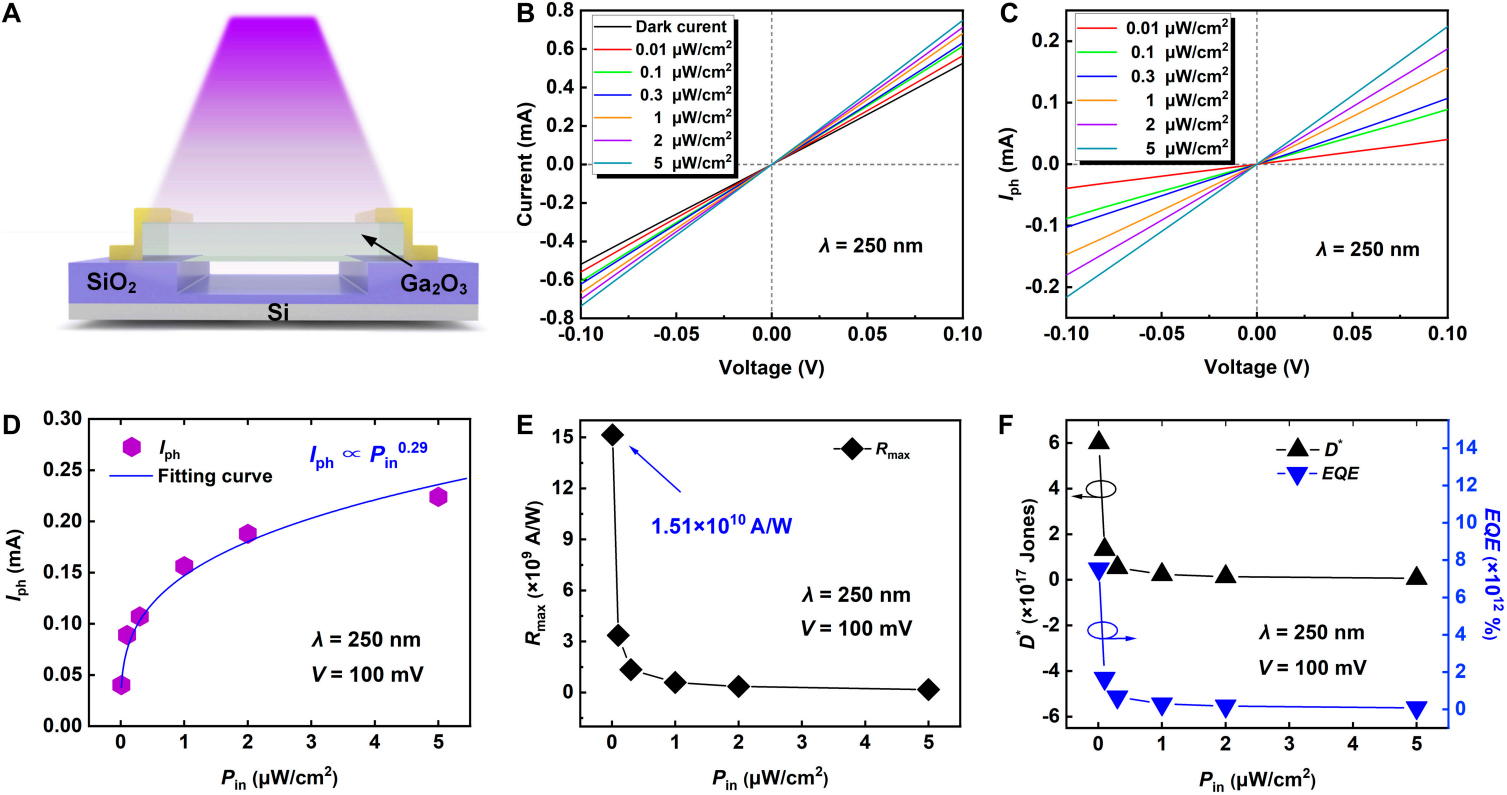
The photoelectrical properties of the fabricated Ga_2_O_3_ photodetector. (A) Illumination method of the DUV light source during the photoelectrical testing of the device. (B) *I*–*V* characteristics of the Ga_2_O_3_ photodetector under different *P*_in_ values in a range of 0 to 5 μW/cm^2^ at a wavelength of 250 nm. (C) Dependence of *I*_ph_ on voltage under different illumination *P*_in_. (D) *I*_ph_ as a function of *P*_in_. (E) Corresponding *R*_max_ dependence of *P*_in_ under 250-nm illumination. (F) $D^{*}$ and *EQE* values versus *P*_in_ under 250-nm illumination.

Then, the electrical and photoelectrical properties of the fabricated Ga_2_O_3_ photodetector with a suspended structure were systematically measured. Figure 3A illustrates the experimental setup for photoelectrical testing, where DUV light is perpendicularly directed onto the surface of the device, covering the entire Ga_2_O_3_ channel. Figure 3B displays the current–voltage (*I*–*V*) characteristics of the Ga_2_O_3_ photodetector under different *P*_in_ conditions, ranging from 0 to 5 μW/cm^2^ at a wavelength of 250 nm. Under dark conditions, the *I*–*V* curve displays linearity and symmetry, indicating the formation of good ohmic contacts between the electrodes and the Ga_2_O_3_ channel. This was achieved by using a Ti/Al/Ni/Au electrode stack, followed by rapid thermal annealing at 470 °C for 60 s. During the annealing process, Al atoms diffuse rapidly into the Ti layer, forming a Ti–Al intermetallic phase with a low work function, which creates chemically stable contacts and induces oxygen vacancies at the Ga_2_O_3_/electrode interface [22]. These oxygen vacancies promote ohmic contact formation and greatly reduce the contact resistance, causing the device to exhibit a high dark current of 0.52 mA at *V* = 0.1 V. In terms of photoelectrical performance, the current increases with higher *P*_in_ under 250-nm illumination, as evident in Fig. 3B, demonstrating the high DUV sensitivity of the Ga_2_O_3_ device. Notably, the device shows substantial photocurrent (*I*_ph_), where *I*_ph_ represents the photocurrent generated under each illumination intensity even under very weak illumination conditions, such as *P*_in_ = 0.01 mW/cm^2^. The extracted values *I*_ph_ for various *P*_in_ levels are plotted in Fig. 3C, where the Ga_2_O_3_ photodetector generates *I*_ph_ values of 40, 89, 107, 156, 188, and 224 μA at *V* = 0.1 V for *P*_in_ values of 0.01, 0.1, 0.3, 1, 2, and 5 μW/cm^2^, respectively. The increase in *I*_ph_ with increasing *P*_in_ is attributed to the excitation of more photogenerated carriers, which enhances photoconductivity [23]. To investigate the relationship between *I*_ph_ and *P*_in_, Fig. 3D presents a plot of *I*_ph_ versus *P*_in_ at *V* = 0.1 V, fitted using the power law formula of *I*_ph_ ∝ ${\mathrm{AP}}_{{\mathrm{in}}^{\alpha }}$, where *A* is a constant and α is the index of power law. Ideally, α should be equal to 1, which indicates a linear response. However, in this work, the obtained α value is 0.29, which is considerably less than 1, confirming the presence of electron-hole recombination centers caused by defects in the Ga_2_O_3_ channel l, as previously confirmed by PL measurements [24]. Although this sublinear response indicates some recombination losses, it offers a notable advantage in terms of higher sensitivity at low light intensities, where even small increases in incident power result in relatively larger changes in photocurrent. This characteristic makes the Ga_2_O_3_ photodetector particularly suitable for detecting weak ultraviolet signals, demonstrating high responsivity under ultra-low illumination conditions, which is critical for applications requiring enhanced sensitivity in low-light environments.

To further quantify the optoelectronic performance, key metrics such as *R*, $D^{*}$, and *EQE* are calculated. The responsivity *R*, which represents the photocurrent generated per unit incident light power on the effective area, was determined using the following equation [25]:

$$R=\frac{I_{\text{ph}}}{P_{\text{in}}\cdot S},$$ (1)

where *I*_ph_ is the photogenerated current, *P*_in_ is the light power density, and *S* is the effective area of the Ga_2_O_3_ photodetector. Figure 3E plots the maximum responsivity (*R*_max_) for different *P*_in_ conditions, showing a clear trend where *R*_max_ decreases with increasing *P*_in_. This decline is likely due to the saturation of photogenerated carriers at higher light intensities, reflecting the complex interplay of generation, separation, and transport processes of photocarriers [26]. The Ga_2_O_3_ photodetector achieves *R* values of 1.51 × 10^10^, 3.36 × 10^9^, 1.35 × 10^9^, 5.89 × 10^8^, 3.54 × 10^8^, and 1.69 × 10^8^ A/W for *P*_in_ values of 0.01, 0.1, 0.3, 1, 2, and 5 μW/cm^2^, respectively. Remarkably, the device achieves an exceptionally high *R* of 1.51 × 10^10^ A/W under very low illumination (*P*_in_ of 0.01 μW/cm^2^), which is attributed to the suspended structure. This design minimizes direct contact between the Ga_2_O_3_ channel and the substrate, thereby reducing carrier trapping by interface defects and improving carrier collection efficiency [27].

Another parameter detectivity $D^{*}$, another key parameter, is used to describe the sensitivity of the device relative to the noise level, assuming that shot noise dominates. $D^{*}$ is calculated using the following equation [25]

D∗=S12·R2e·Idark12, (2)

where *e* is the carrier charge (1.6 × 10^−19^ C) and *I*_dark_ is the dark current. As shown in Fig. 3E, $D^{*}$ reaches 6.01 × 10^17^ Jones under the lowest *P*_in_ condition of 0.01 μW/cm^2^. Even under higher *P*_in_, the device maintains $D^{*}$ values in the order of 10^16^ Jones, indicating excellent signal detection capabilities with minimal noise interference. Finally, the external quantum efficiency *EQE* of the Ga_2_O_3_ photodetector was calculated, which represents the ratio of the number of converting absorbed photons to electrons and the total number of excitation photons. The *EQE* can be extracted from the equation [25]:

$$E\mathrm{QE}=\frac{\mathrm{hc}}{\mathrm{e\lambda}}\cdot R,$$ (3)

where *h* is the Planck’s constant (6.626 × 10^−34^ Js), *c* is the speed of light (3.0 × 10^8^ m/s), *e* is the elementary charge, and *λ* is the illumination wavelength (250 nm). As shown in Fig. 3F, the maximum *EQE* is as high as 7.53 × 10^12^ % under *P*_in_ = 0.01 μW/cm^2^ at *V* = 0.1 V. However, as *P*_in_ increases, the *EQE* gradually decreases, likely due to the increased recombination rate of photogenerated carriers at higher light intensities. In summary, the Ga_2_O_3_ photodetector with a suspended structure exhibits excellent photoelectrical performance, particularly in terms of high DUV sensitivity and the ability to detect weak signals. These characteristics, combined with its superior structural properties, make the device highly promising for DUV photodetection applications.

**Fig. 4.**
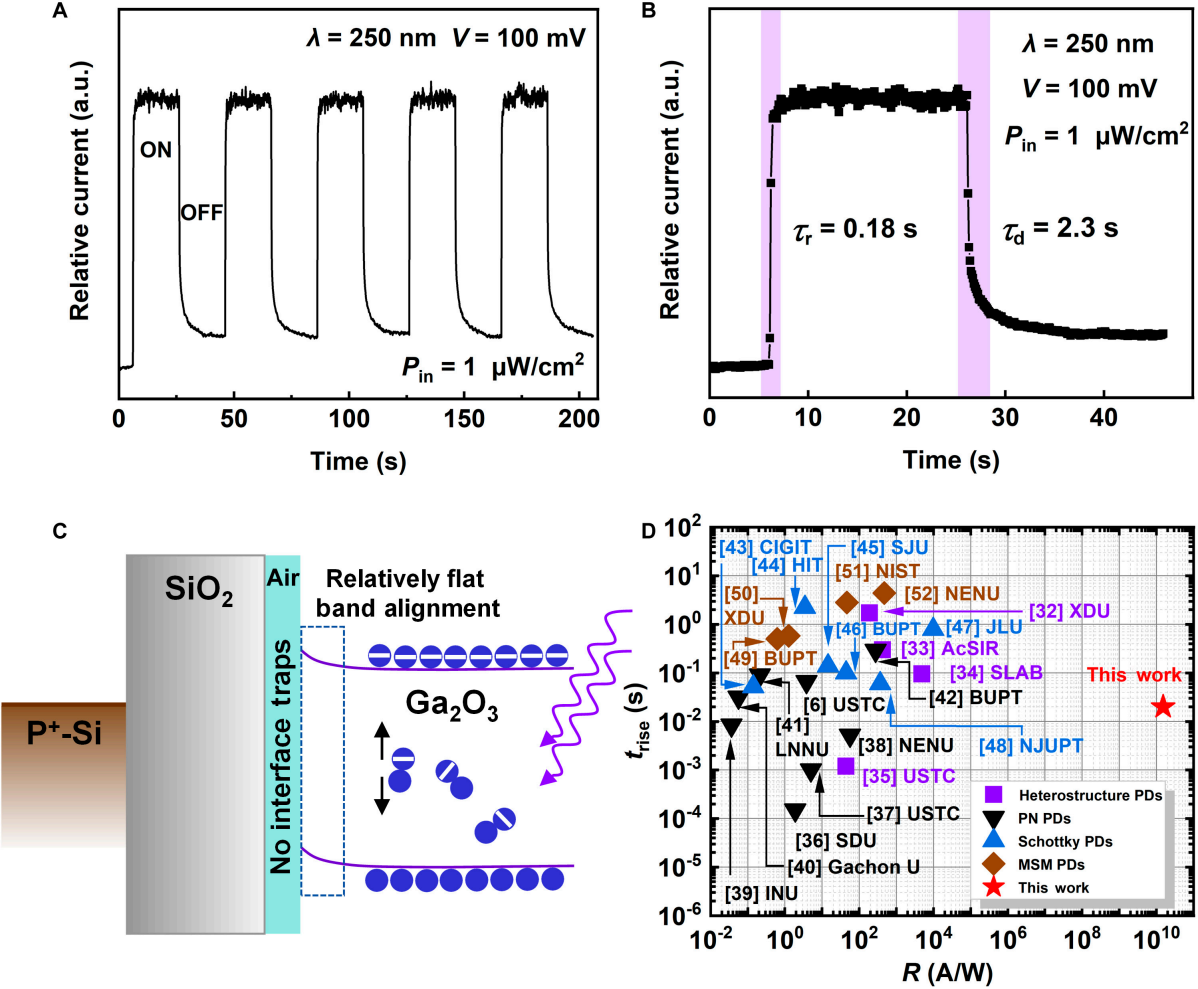
The response time properties of fabricated Ga_2_O_3_ photodetectors. (A) Transient photoresponse properties of device by periodically switching the light source under the illumination *P*_in_ of 0.01 μW/cm^2^ at *V* = 0.1 V. (B) Enlarged view of the rise and decay edges of the curve in (A). (C) Band diagram of Ga_2_O_3_ device under 250-nm illumination. (D) Benchmarks of the responsivity and response time of Ga_2_O_3_ photodetectors.

Following the analysis of the device’s overall photoelectrical performance, the transient response behavior was investigated to further understand the dynamic characteristics of the Ga_2_O_3_ photodetector. Figure 4A shows the transient photoresponse properties of the Ga_2_O_3_ photodetector with a suspended structure under 250-nm illumination, with an incident *P*_in_ of 0.01 μW/cm^2^ at *V* = 0.1 V. The illumination was cycled on and off every 20 s, with a total cycle period of 40 s. The device exhibited stable and repeatable ON-OFF switching behavior, indicating a strong and consistent photoresponse. To evaluate the *τ*_r_ and the decay time (*τ*_d_) of the Ga_2_O_3_ photodetector with a suspended structure, Fig. 4B provides a detailed view of the rise and decay edges of the photoresponse curve from Fig. 4A. The rise time *τ*_r_, defined as the time required for the photocurrent to increase from 10% to 90% of its maximum value after the light is turned on, was measured at 0.18 s. Similarly, the decay time *τ*_d_, defined as the time it takes for the photocurrent to decrease from 90% to 10% of its maximum value after the light is turned off, was measured at 2.3 s. These values indicate a fast response to illumination changes, making the device highly suitable for real-time DUV detection applications. To gain deeper insight into the enhanced photoelectrical properties attributed to the suspended structure, Fig. 4C presents the energy band diagram of the Ga_2_O_3_ device under 250-nm illumination. In the suspended structure of the Ga_2_O_3_ photodetector, the design minimizes direct contact between the Ga_2_O_3_ channel and the substrate. This architectural approach plays a crucial role in enhancing the device’s performance by reducing interface defects and suppressing carrier recombination that typically occurs at the interface in conventional devices. Interface defects commonly introduce deep-level traps that act as recombination centers for photogenerated carriers, increasing dark current and degrading detectivity [28]. In contrast, the suspended Ga_2_O_3_ structure effectively lowers the density of these defects, thereby reducing the dark current and enhancing the signal-to-noise ratio, which is critical for high-performance photodetectors [27]. Moreover, the suspended structure greatly improves carrier transport and collection efficiency. In conventional Ga_2_O_3_ devices, band bending near the interface can hinder carrier mobility by creating potential barriers that trap carriers, leading to recombination. In the suspended structure, as illustrated in the energy band diagram in Fig. 4C, this band bending is largely avoided, leading to a relatively flat band alignment [29]. The flat band configuration allows photogenerated carriers to experience minimal energy loss during transport while also reducing the bandgap distortion caused by substrate-induced thermal noise or interface scattering. As a result, the photogenerated electrons and holes migrate more efficiently toward their respective electrodes, thereby enhancing the overall carrier mobility and improving the device’s responsivity. When exposed to 250-nm DUV illumination, high-energy photons are absorbed by the Ga_2_O_3_ material, generating electron-hole pairs. In the suspended Ga_2_O_3_ device, the absence of interface traps drastically reduces recombination losses, allowing a larger fraction of the photogenerated carriers to contribute to the photocurrent. The suspended structure creates a strong electric field between the electrodes, efficiently separating the photogenerated electrons and holes and driving them toward the anode and cathode, respectively. This reduction in recombination, coupled with enhanced carrier transport, directly leads to the fast rise time *τ*_r_ of 0.18 s observed in Fig. 4B [30]. Furthermore, the suspended structure enhances light absorption efficiency by preventing direct interaction between the Ga_2_O_3_ channel and the substrate. This design minimizes parasitic absorption losses, which are common in substrate-supported devices, thereby ensuring that more incident photons are available to generate electron-hole pairs. The increased absorption of photons further boosts the photocurrent and enhances the device’s responsivity [31]. The combined effects of efficient carrier separation, reduced recombination, and enhanced light absorption result in remarkable improvements in both responsivity and response time, thereby greatly enhancing the overall photoelectric performance of the device. Figure 4D provides a benchmark comparison of the responsivity and response time of Ga_2_O_3_ photodetectors reported in the literature [6,32–52]. Typically, there is a trade-off between responsivity and response time in photodetector design, where improvements in one parameter often come at the cost of the other. However, the suspended Ga_2_O_3_ photodetector presented in this work achieves both ultra-high responsivity and rapid response times, surpassing many of the previously reported devices. This highlights the potential of substrate-free or suspended device structures in achieving high-performance Ga_2_O_3_ photodetectors for next-generation DUV sensing applications.

## Conclusion

In summary, high-performance Ga_2_O_3_ photodetectors with a suspended structure were successfully demonstrated for the first time, showcasing exceptional sensitivity to ultra-weak DUV light, with the ability to detect illumination levels as low as 0.01 μW/cm^2^. Notably, the photodetector achieves a high responsivity of 1.51 × 10^10^ A/W, a sensitive detectivity of 6.01 × 10^17^ Jones, a large external quantum efficiency of 7.53 × 10^12^ %, and a fast rise time of 180 ms under 0.01 μW/cm^2^ illumination at *V* = 0.1 V. These remarkable improvements are primarily attributed to the reduction of interface defects, improved carrier transport, efficient carrier separation, and enhanced light absorption enabled by the suspended structure. This research offers a viable alternative for designing and optimizing high-performance Ga_2_O_3_ solar-blind photodetectors, advancing the field of optoelectronics.

## Materials and Methods

### Device fabrication

First, a 110-nm SiO_2_/p^++^-Si substrate was prepared and cleaned using isopropyl alcohol, acetone, and deionized water, followed by drying with a nitrogen spray gun. Then, trenches intended for the substrate-free structure were patterned using an electron-beam lithography (EBL) process. The trenches were etched using CF_4_ + Ar plasma in a RIE setup with the following parameters: CF₄ and Ar gas flow rates of 25 and 5 sccm (standard cubic centimeters per minute), respectively, a pressure of 10 mtorr, a radio-frequency power of 150 W, and an etching time of 25 s. After this, mechanically exfoliated Ga_2_O_3_ was transferred onto the fabricated trenches using a 2D transfer platform, ensuring accurate alignment and placement. (The Raman spectrum of Ga_2_O_3_ nanosheet is displayed in Fig. S2.) Electrode regions were then defined by an additional EBL process, and a multilayer stack of Ti/Al/Ni/Au (20/100/60/80 nm) was deposited using electron-beam evaporation equipment, followed by a standard lift-off process in an acetone solution. Finally, the manufactured Ga_2_O_3_ device was annealed at 470 °C for 60 s in a high pure nitrogen atmosphere using rapid thermal annealing equipment to ensure the formation of good ohmic contact between the Ga_2_O_3_ channel and the electrodes.

### Characterizations and measurements

The depth of the suspended trench and the thickness of the Ga_2_O_3_ channel were measured using AFM equipment (Oxford MFP-3D). The crystalline structure of the Ga_2_O_3_ material was analyzed using GIXRD with an x-ray wavelength of 0.6887 Å. A 2D-GIXRD pattern was acquired by a PILATUS detector at positioned approximately 263 mm from the Ga_2_O_3_ sample, with an exposure time of 20 s. The optical properties of the Ga_2_O_3_ material were examined through Raman spectroscopy (LabRAM HR800, Horiba Jobin Yvon) with a 532-nm laser excitation wavelength and PL spectroscopy (FLS980 D2D2, Edinburg) with a 325-nm laser excitation wavelength. The high-resolution structural analysis of the Ga_2_O_3_ material was characterized by using HR-TEM (Talos F200X). Optical transmittance characterization of Ga_2_O_3_ samples was determined using a UV-visible spectrophotometer (UV-3100). The electrical and optoelectronic properties of the Ga_2_O_3_ photodetector were measured using a semiconductor analyzer (Agilent B1500A), with a deuterium lamp (THORLABS SLS204) serving as the DUV source. The calibration of the incident *P*_in_ was implemented by an optical power meter (THORLABS S120VC).

## Data Availability

Supplementary materials contain additional data. All data needed to evaluate the conclusions in the paper are present in the paper or the Supplementary Materials.
