# Supplementary material for: Ga2O3 Solar-Blind Deep-Ultraviolet Photodetectors with a Suspended Structure for High Responsivity and High-Speed Applications
Source: Research (Wash D C). 2024 Dec 11;7:0546. doi: 10.34133/research.0546 (PMC11632154; doi:10.34133/research.0546)
Supplement: Supplementary 1 — Figs. S1 and S2 [file research.0546.f1.docx]

Supporting Information

**Ga_2_O_3_ solar-blind deep-ultraviolet photodetectors with a suspended structure for high responsivity and high-speed applications**

**Xiaoxi Li^1,2^, Zhifan Wu^1^, Yuan Fang^1^, Shuqi Huang^1^, Cizhe Fang^1,2^, Yibo Wang^1,2^, Xiangyu Zeng^1,2^, Ying-Guo Yang^3,4*^, Yue Hao^1,2^, Yan Liu^1,2^, and Genquan Han^1,2*^**

^1^Hangzhou Institute of Technology, Xidian University, Hangzhou, 311200, China

^2^School of Microelectronics, Xidian University, Xi’an, 710071, China

^3^State Key Laboratory of ASIC and System, Shanghai Institute of Intelligent Electronics & Systems, School of Microelectronics, Fudan University, Shanghai 200433, China

^4^Shanghai Synchrotron Radiation Facility (SSRF), Shanghai Institute of Applied Physics & Shanghai Advanced Research Institute, Chinese Academy of Sciences, Shanghai 201208, China.

E-mail: yangyingguo@fudan.edu.cn, gqhan@xidian.edu.cn.


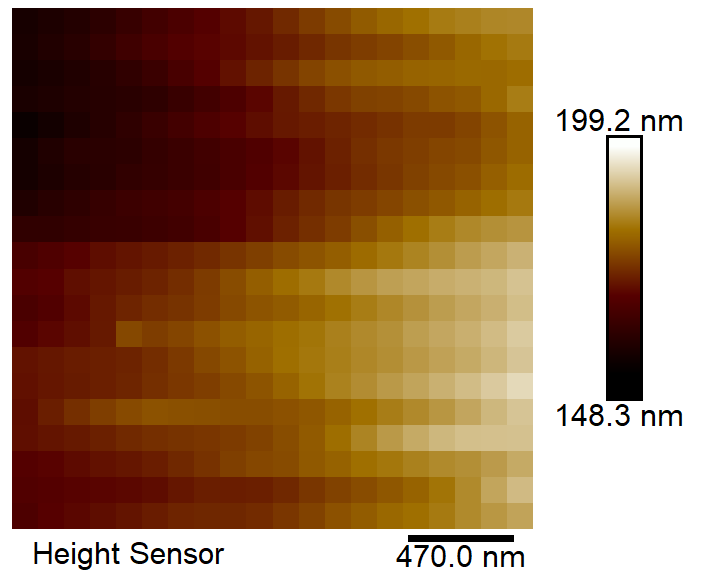


Fig. S1. The surface morphology of Ga_2_O_3_ channel.


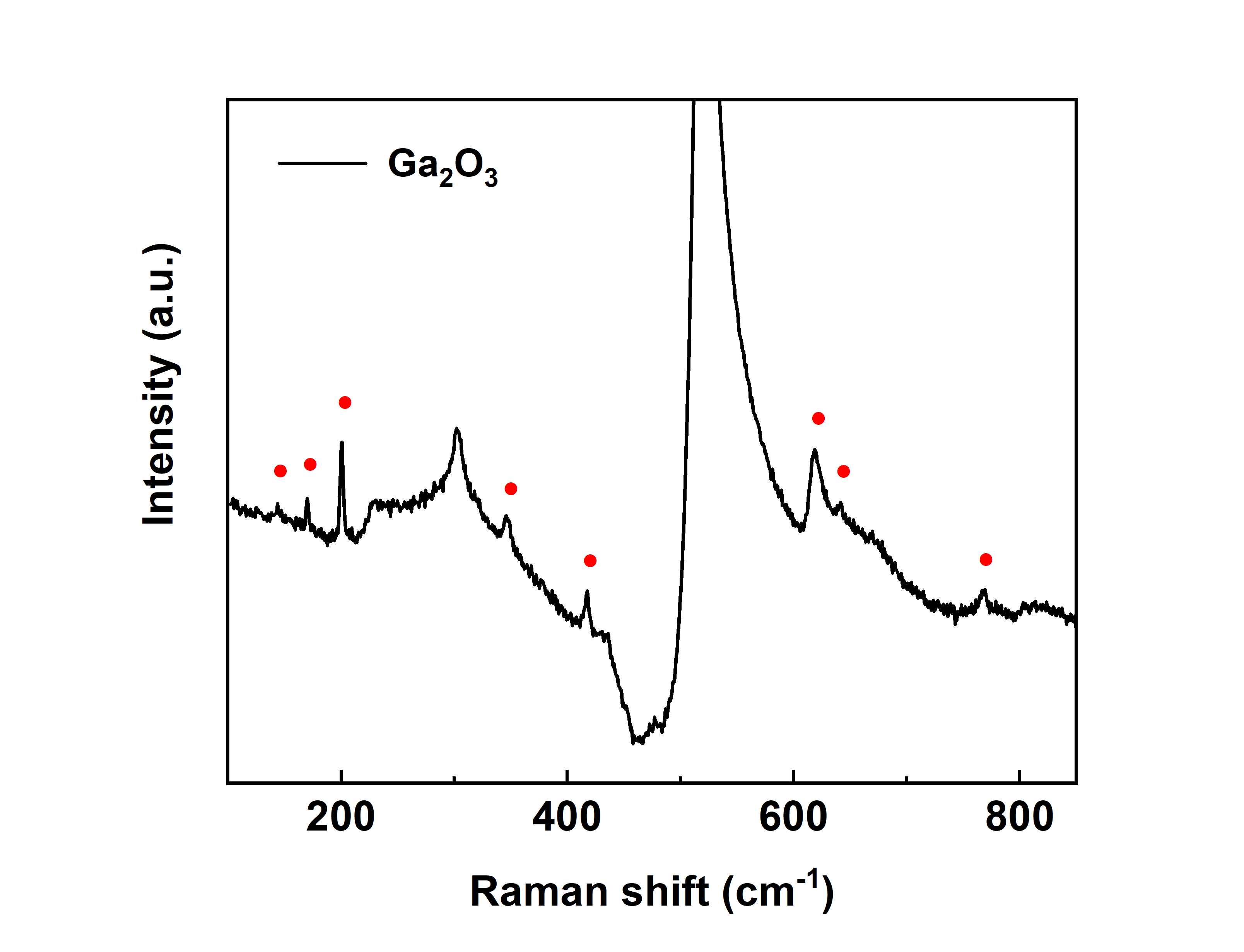


Fig. S2. Raman spectrum of exfoliated Ga_2_O_3_ channel.
